# Bifascicular Block Associated With Myocardial Infarction: A Marker of Proximal Left Anterior Descending Artery Occlusion Confirmed by the Artificial Intelligence-Based Smartphone App Queen of Hearts

**DOI:** 10.7759/cureus.97853

**Published:** 2025-11-26

**Authors:** Lucio Giuseppe Granata, Francesco Russo, Simona Giubilato, Marilena Scarabelli, Francesco Amico

**Affiliations:** 1 Department of Clinical and Experimental Medicine, Cardiology Unit, University Hospital Polyclinic G. Martino, Messina, ITA; 2 Cardiology Division, Cannizzaro Hospital, Catania, ITA

**Keywords:** artificial intelligence, bifascicular block, heart failure with reduced ejection fraction (hfref), left anterior descending artery, left anterior fascicular block, occlusive myocardial infarction, queen of hearts, right bundle branch block, st-elevation myocardial infarction (stemi), stemi equivalent

## Abstract

In a clinical context highly suggestive of acute coronary syndrome (ACS), the presence of a bifascicular block (BFB) should be considered a potential ST-elevation myocardial infarction (STEMI) equivalent. A right bundle branch block (RBBB) may obscure accurate assessment of the ST segment, potentially masking ischemic changes. Therefore, in patients with suspected ACS, BFB (RBBB and left anterior fascicular block), whether new or pre-existing, should be managed as a high-risk finding and treated according to STEMI protocols if clinical suspicion is strong. We report the case of a man in his 70s with multiple cardiovascular risk factors who was admitted for chest pain and hypotension. The ECG revealed a new (or presumably new) BFB, consisting of RBBB and left anterior fascicular block, associated with QRS-concordant anterior ST-segment elevation extending to lateral leads. Urgent coronary angiography demonstrated a proximal left anterior descending (LAD) artery subocclusion, which was successfully treated with primary percutaneous coronary intervention and drug-eluting stent implantation. The artificial intelligence (AI)-based smartphone application *Queen of Hearts* (PMcardio) correctly interpreted this ECG pattern as a STEMI-equivalent, accurately identifying rhythm, conduction abnormalities, and predicting a reduced left ventricular ejection fraction. This case underscores the importance, within the appropriate clinical context, of early recognition of BFB as a STEMI-equivalent or occlusive myocardial infarction pattern, typically associated with proximal LAD occlusion, extensive myocardial injury, and poor prognosis. At the same time, it provides anecdotal evidence supporting the reliability and clinical utility of the AI-based *Queen of Hearts* app in ACS management.

## Introduction

The 2023 European Society of Cardiology (ESC) guidelines for acute coronary syndrome (ACS) define ST-elevation myocardial infarction (STEMI) as the presence of new ST-segment elevation (STE), measured at the J-point (the junction between the QRS complex and the ST segment), in at least two contiguous leads (including V3R and V4R): ≥2.5 mm in men <40 years old, ≥2.0 mm in men ≥40 years old, or ≥1.5 mm in women (regardless of age) in leads V2-V3, and/or ≥1 mm in other leads, in the absence of left ventricular hypertrophy or left bundle branch block [1]. STEMI usually reflects a large heart attack caused by a sudden, complete blockage of a coronary artery, leading to full-thickness myocardial injury.

However, due to the well-recognized limitations of these criteria, particularly their low sensitivity and imperfect performance as a surrogate marker of acute coronary occlusion, a paradigm shift toward an occlusive myocardial infarction (OMI)-based ECG diagnostic approach has been strongly advocated [2]. OMI refers to myocardial infarction caused by a fully or nearly fully occluded coronary artery that requires urgent reperfusion, even when classic ST-elevation criteria are not met.

In this context, the supplementary material of the ESC guidelines describes STEMI-equivalent patterns, ECG findings that do not display overt STE but still reflect total or subtotal acute coronary occlusion, thus requiring emergent reperfusion therapy. The document also emphasizes that, in the presence of right bundle branch block (RBBB), STE indicates STEMI, whereas ST depression in leads I, aVL, and V5-V6 is more consistent with NSTEMI [1,3]. Certain conduction abnormalities can mask or modify ST-segment changes, making the recognition of high-risk patterns more challenging.

Despite these clarifications, current ESC guidelines do not specifically address the diagnosis, management, or prognostic implications of bifascicular block (BFB), defined as RBBB combined with left anterior fascicular block (LAFB), as a potential STEMI-equivalent or OMI marker in patients presenting with suspected myocardial ischaemia.

BFB may complicate acute myocardial infarction (AMI), typically reflecting proximal left anterior descending (LAD) coronary artery severe obstruction, and is associated with poor prognosis due to extensive myocardial injury, heart failure with reduced ejection fraction, and other adverse outcomes. Early percutaneous coronary intervention (PCI) in this context significantly improves prognosis and reduces mortality [4]. Therefore, in the setting of suspected ACS, the presence of a new or presumably new BFB should be promptly recognized by both cardiologists and emergency physicians as a life-threatening, time-critical, high-risk ECG pattern indicative of OMI, warranting immediate reperfusion therapy [5].

Recently, artificial intelligence (AI)-powered smartphone applications have emerged as promising tools in this field. The *Queen of Hearts* app by PMcardio is an advanced AI-based ECG interpretation model that, through three simple steps, aims to move beyond traditional STE assessment to identify STEMI-equivalent/OMI patterns, thus supporting rapid, accurate clinical decision-making and reducing both false-negative and false-positive STEMI diagnoses [6]. These tools analyze digital ECGs and provide automated interpretations to assist clinicians, but they are intended to complement, not replace, expert ECG reading and clinical judgment.

We present the case of a man in his 70s who experienced an AMI-associated BFB, acting as a STEMI-equivalent/OMI-specific sign, highlighting its association with extensive myocardial injury and poor prognosis due to subsequent heart failure, even after optimal reperfusion. Additionally, we provide anecdotal evidence of how the *Queen of Hearts* app supported the diagnostic process and even predicted left ventricular dysfunction in this clinical scenario.

## Case presentation

**Figure 1:**
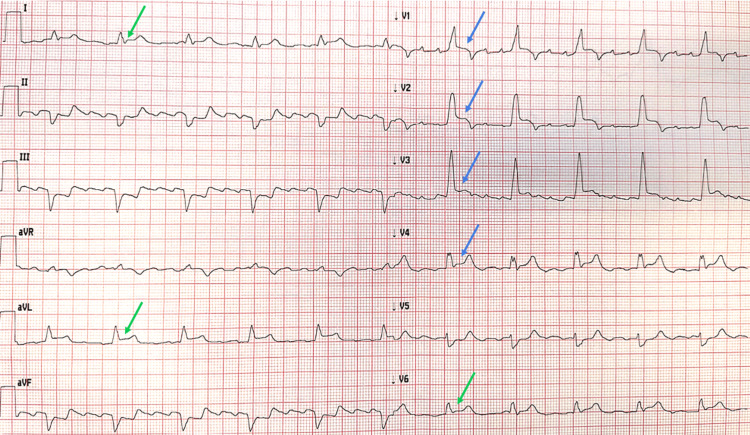
Anterolateral STEMI with atrial flutter and bifascicular block. Initial ECG demonstrating anterolateral STEMI with atrial flutter (4:1 conduction) and BFB (RBBB and LAFB). ST-segment elevation concordant with the terminal positive portion of the QRS complex is present in leads V1–V4 (blue arrows), along with lateral ST elevation (green arrows). STEMI = ST-elevation myocardial infarction; BFB = bifascicular block; RBBB = right bundle branch block; LAFB = left anterior fascicular block

A 77-year-old man was evaluated by the Emergency Medical Service for intermittent chest pain that had begun three days earlier and became continuous overnight, unresponsive to self-administered sublingual nitrates. The first ECG (Figure 1) was recorded by the emergency physician and transmitted to our Cardiac Intensive Care Unit via the wireless STEMI network for evaluation. It demonstrated typical atrial flutter with 4:1 atrioventricular conduction (mean heart rate ≈ 70 bpm), bifascicular block (complete RBBB and LAFB), and QRS-concordant STE in anterior leads (V1-V4, blue arrows) with additional STE in lateral leads (I, aVL, V6, green arrows). Inferior leads (II, III, aVF) displayed reciprocal changes.

A diagnosis of anterior STEMI with lateral extension in the context of new or presumably new BFB was made, and the patient was transferred to our Cardiology Division for emergent coronary angiography.

The patient’s medical history included smoking, hypertension, dyslipidemia, chronic kidney disease, obstructive sleep apnea, aorto-femoral bypass, non-critical carotid disease, ischemic stroke, previous lung cancer resection, and unspecified arrhythmia treated with a direct oral anticoagulant. He had been recently hospitalized for pneumonia, acute kidney injury, hypomagnesemia, and hypocalcemia. His home medications included bisoprolol 1.25 mg, apixaban 2.5 mg b.i.d., magnesium, calcium, epoetin alfa, and amlodipine 5 mg.

On admission, he reported crushing chest pain with cold sweating. He was hypotensive (blood pressure of 73/53 mmHg), pale, and normoxemic (SpO₂ of 96% on room air). Physical examination revealed a grade 2/6 systolic apical murmur, bibasilar crackles on chest auscultation, and mild peripheral edema. The catheterization laboratory was immediately activated.

**Figure 2:**
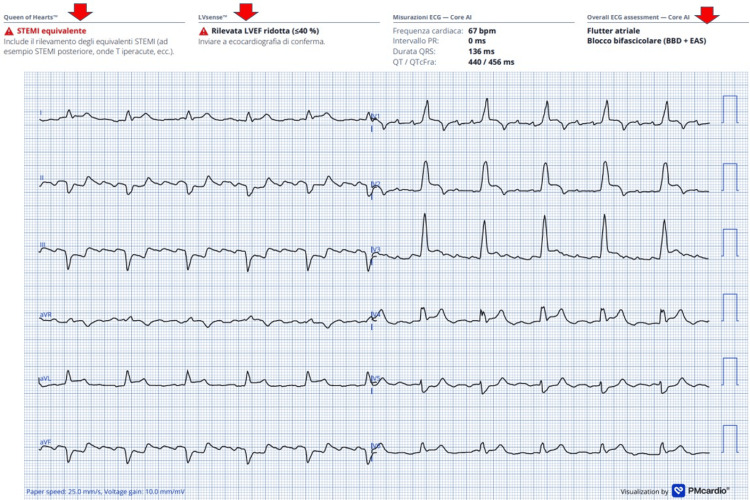
Initial ECG: Queen of Hearts (PMcardio) analysis. The AI-based ECG interpretation correctly identified the STEMI-equivalent pattern, rhythm (atrial flutter), conduction abnormalities (RBBB + LAFB), and a predicted EF <40%. ECG = electrocardiogram; AI = artificial intelligence; STEMI = ST-elevation myocardial infarction; RBBB = right bundle branch block; LAFB = left anterior fascicular block; EF = ejection fraction

Meanwhile, the first ECG was also processed using the AI-based smartphone application *Queen of Hearts* (PMcardio), capable of detecting STEMI/STEMI-equivalent patterns (Figure 2). As shown in Figure 2 (red arrows), the app correctly identified a STEMI-equivalent pattern, detected atrial flutter, recognized the bifascicular block (RBBB + LAFB), and predicted a reduced ejection fraction (EF <40%). The AI tool served only as confirmatory support and did not influence the decision-making strategy, as the invasive strategy had already been determined.

**Figure 3:**
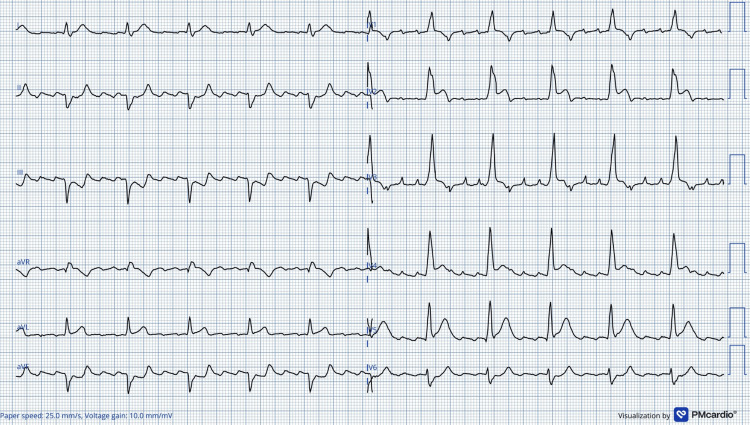
Second ECG. Visualization by Queen of Hearts (PMcardio). Anterolateral STEMI, atrial flutter, BFB with further progression of QRS-concordant STE (V2–V5) compared with the initial tracing. STEMI = ST-elevation myocardial infarction; BFB = bifascicular block; STE = ST-segment elevation

A second ECG (Figure 3) confirmed the previous findings and showed progression of STE in leads V2-V5. The *Queen of Hearts* (PMcardio) app again correctly interpreted the tracing as STEMI.

**Figure 4:**
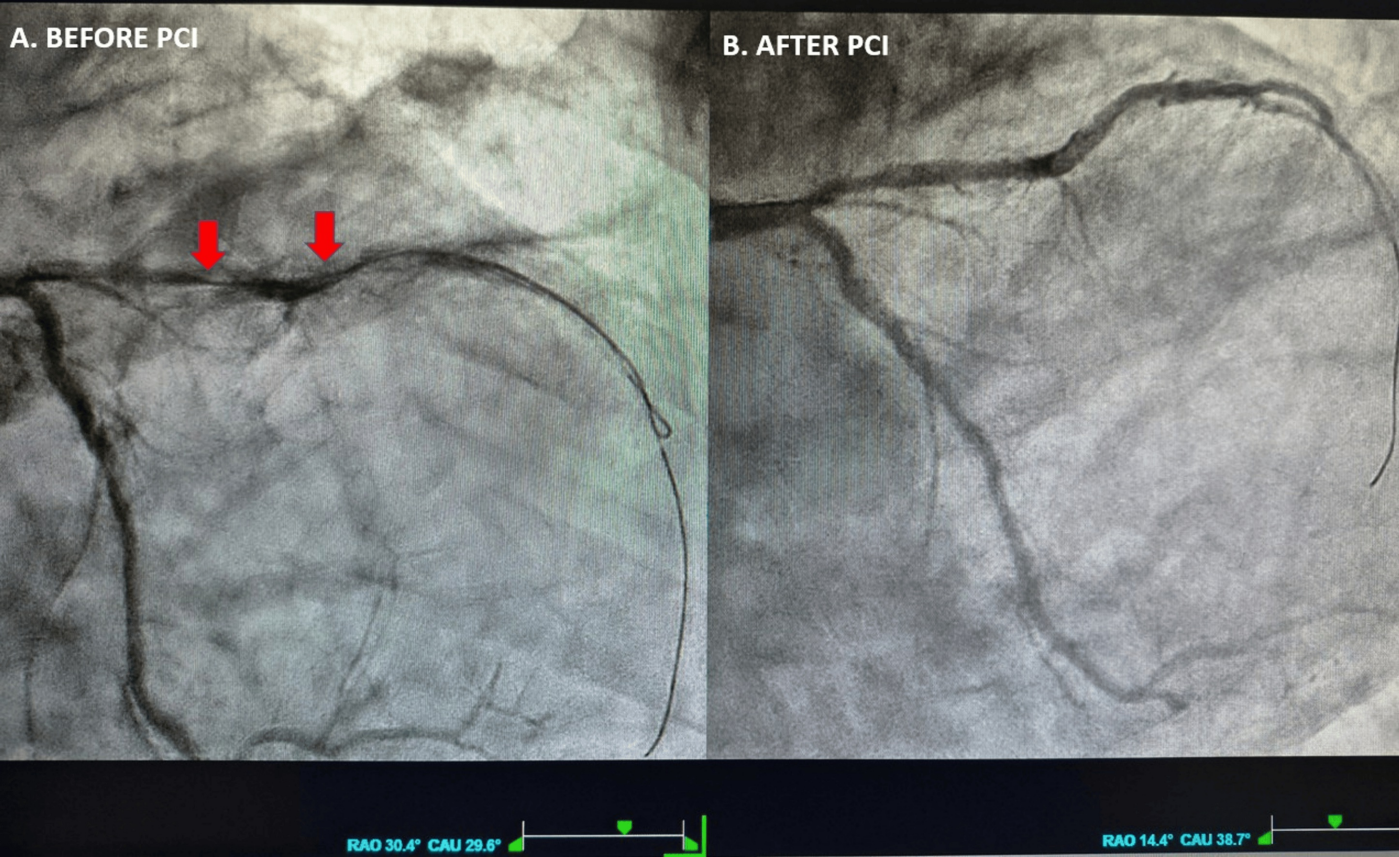
Coronary angiography. Calcified proximal LAD artery occlusion before (A) and after (B) pPCI. LAD = left anterior descending artery; pPCI = primary percutaneous coronary intervention

Coronary angiography revealed a long, severely calcified, near-occlusive stenosis of the left anterior descending (LAD) artery (Figure 4A, red arrows; Video 1, left panel), which was treated with primary percutaneous coronary intervention (pPCI) and drug-eluting stent implantation, achieving a good final angiographic result (Thrombolysis in Myocardial Infarction flow grade 3) (Figure 4B; Video 1, right panel). The left circumflex artery and the right coronary artery showed mild, non-obstructive atherosclerosis.

**Video 1:** Coronary angiography before (left side) and during (right side) pPCI. pPCI = primary percutaneous coronary intervention

**Figure 5:**
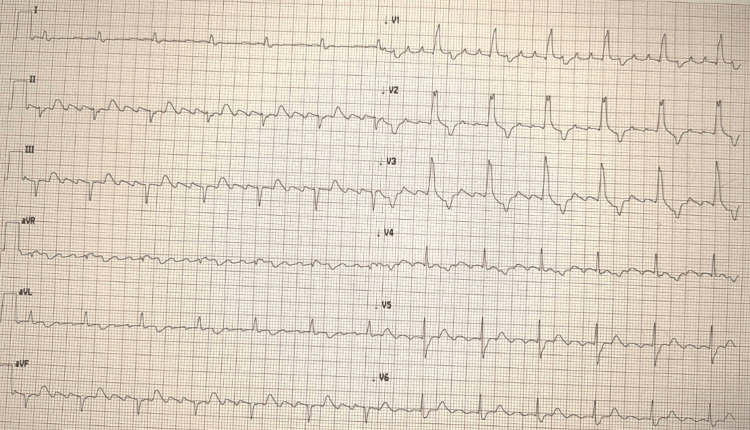
ECG recorded the following day after pPCI. Following-day ECG showing persistent BFB, but, unlike the previous tracing, repolarization changes secondary to RBBB are now evident; post-reperfusion negative T waves are also visible in V4 and aVL. ECG = electrocardiogram; pPCI = primary percutaneous coronary intervention; BFB = bifascicular block; RBBB = right bundle branch block

After reperfusion, the BFB persisted, but STE resolved, and QRS-discordant negative T waves appeared in V1-V3, typical of RBBB-related repolarization, persisting in the following days (Figure 5).

The most relevant laboratory findings are reported in Table 1. The patient presented with moderate anemia and moderate-to-severe chronic kidney disease, with an estimated glomerular filtration rate of 32 mL/minute/1.73 m² (stage IIIb). High-sensitivity troponin I and N-terminal pro-B-type natriuretic peptide levels were elevated but gradually decreased before discharge. Low-density lipoprotein cholesterol levels remained above the recommended target (<40 mg/dL) for patients with multiple cardiovascular events [1].

**Table 1:** Relevant laboratory values.

Laboratory parameter	Admission	Reference range	Unit
Hemoglobin	9.7	13–17	g/dL
High-sensitivity troponin-I	16,894	<12	ng/L
N-terminal pro-B-type natriuretic peptide	23,714	0–450	pg/mL
Glycated hemoglobin	36	<46	mmol/mol
Creatinine	1.96	0.6–1.1	mg/dL
Estimated glomerular filtration rate	32	90–120	mL/minute
Sodium	139	135–145	mEq/L
Potassium	4	3.5–5	mEq/L
Glucose	123	60–100	mg/dL
Low-density lipoprotein-cholesterol	65	(<110)	mg/dL

Echocardiography the day after pPCI (Video 2) showed apical akinesia, anterior and anteroseptal hypokinesia, moderate left ventricular systolic dysfunction (EF: 38%), normal right ventricular function, biatrial enlargement, and moderate ischemic mitral regurgitation, thus confirming the *Queen of Hearts* app’s predicted EF value (via the LV Sense function).

**Video 2:** Echocardiography the day after percutaneous coronary intervention. Apical, anterior, and anteroseptal wall motion abnormalities are present, associated with a reduced left ventricular ejection fraction (<40%). There is also evidence of significant ischemic mitral regurgitation.

In-hospital therapy included acetylsalicylic acid (discontinued seven days after pPCI), clopidogrel, apixaban reduced dose (2.5 mg bid), proton-pump inhibitor, ezetimibe/atorvastatin, furosemide, SGLT2 inhibitor, ramipril, and potassium canrenoate. The clinical course was uneventful. After eight days, the patient was discharged in good clinical condition on optimized heart failure therapy (the “four pillars”) with instructions to discontinue clopidogrel after 12 months. A one-month follow-up visit was scheduled.

## Discussion

RBBB is defined by a QRS duration >120 ms, an rsR’ (“bunny-ear”) pattern in the anterior precordial leads (V1-V3), and slurred S waves in leads I, aVL, and often V5-V6 [1]. Its prevalence in the general population without structural heart disease is approximately 1%, increasing with age, likely due to degenerative conduction system changes [7].

In the past decade, RBBB in the setting of ACS has been recognized as a potential marker of transmural ischemia, reflecting evolving understanding of conduction abnormalities in ischemic heart disease [8]. Although RBBB generally does not obscure the interpretation of STE, it has important diagnostic and prognostic implications, being associated with AMI in 6.3% of cases (3.2% when combined with LAFB) and occurring even without overt STE in nearly 50% of patients [9]. In-hospital mortality is significantly higher among those with new or presumably new RBBB (18.8%) compared with chronic RBBB (6.4%) and is typically linked to anteroseptal infarction, larger infarct size, cardiogenic shock, complete heart block, and short-term mortality [9-11].

The 2023 ESC ACS guidelines recommend that, in the presence of clinical and ECG signs of myocardial ischemia, both LBBB and RBBB should be considered diagnostic of STEMI, as pre-existing repolarization abnormalities are confounding; this applies irrespective of whether the bundle branch block is new or pre-existing [1]. However, some authors challenge such a broad interpretation, arguing that only RBBB accompanied by ST elevation in leads V1-V3 should prompt emergent coronary angiography, rather than RBBB alone [12].

In RBBB, T waves in right precordial leads are usually discordant with the major terminal portion of the QRS complex-negative in V1-V3 and positive in left precordial leads. The presence of QRS-concordant STE is, therefore, an unequivocal sign of STEMI, as demonstrated in our patient [13].

In most individuals, septal perforator branches of the LAD artery supply both the right bundle branch and the left anterior fascicle; consequently, proximal LAD occlusion should be suspected in ACS presenting with bifascicular block (BFB = RBBB + LAFB) [4,10]. This pattern indicates extensive myocardial injury and correlates with poor prognosis, higher incidence of heart failure, ventricular fibrillation, and increased mortality, even in the era of early reperfusion [4,14]. Left main coronary artery occlusion may also produce this pattern, which is reported in up to 26% of patients with RBBB (mostly with LAFB) admitted for AMI [9,15].

Current ESC guidelines do not provide specific recommendations on the management of conduction abnormalities in this context. Among patients presenting to the emergency department with AMI, the incidence of BFB is estimated at 1.5%, with the highest in-hospital mortality (≈17.5%) among conduction-disturbance subgroups [9].

In a noteworthy case series, McLaren described seven patients with ischemic symptoms and BFB (including one with left posterior fascicular block) and identified several with OMI [16]. He underscored the need to distinguish OMIs involving LAD as the culprit vessel from other mimics in the BFB setting. In particular, he highlighted both the risk of unnecessary catheterization lab activations (due to misinterpreted anterior STE or rate-related repolarization changes in rapid atrial fibrillation) and the risk of missed OMIs arising from different culprit vessels, including the LAD, right coronary artery (with an S1Q3T3 pattern mimicking pulmonary embolism), obtuse marginal branch (posterior myocardial infarction with V3-V4 ST depression and T-wave inversion), or bypass grafts.

In our case, left main coronary artery occlusion is less likely, due to the absence of STE in aVR; the presence of STE in V1-V3, V6, aVL, and D1 leads and BFB (RBBB + LAFB) suggests LAD occlusion, proximal to septal and diagonal branches.

Therefore, BFB in an appropriate clinical context suggestive of ACS should prompt urgent evaluation for acute coronary occlusion and be regarded as a STEMI-equivalent, warranting early invasive management and reperfusion therapy to avoid adverse outcomes [5]. Accurate ECG interpretation is crucial to distinguish true OMI patterns from STEMI mimics and to determine when emergent catheterization is justified.

Recently, AI-enabled smartphone applications such as *Queen of Hearts* (PMcardio) have been developed to assist clinicians in recognizing these patterns. Supported by over 20 clinical studies and two regulatory investigations, this tool aims to reduce false-negative STEMI diagnoses and avoid resource-consuming false-positive activations [6,17]. Across multiple publications involving hundreds of patients, *Queen of Hearts* demonstrated superior specificity, higher diagnostic accuracy, and better performance than conventional STEMI criteria, reducing unnecessary catheterization lab activations and identifying hyperacute T waves. These capabilities suggest potential to minimize missed OMIs and inappropriate cath-lab calls [17]. Furthermore, the app provides rapid estimation of left ventricular ejection fraction directly from a 12-lead ECG, with reported sensitivity of 92.4% and specificity of 88.7%, offering a point-of-care screening method for detecting left ventricular dysfunction [18].

A recent multicenter trial published in JACC: Cardiovascular Interventions (October 2025) assessed the diagnostic accuracy of this AI algorithm versus standard triage ECG interpretation in 1,032 consecutive patients triggering emergent reperfusion protocols at three tertiary PCI centers in the United States [19]. Of these, 601 (58%) had true STEMI and 431 (42%) were STEMI mimics. *Queen of Hearts* identified 92% of true STEMIs compared with 71% for standard-of-care interpretation (demonstrating higher sensitivity) and reduced false-positive catheterization laboratory activation from 42% to 8%, confirming remarkable diagnostic potential.

To further explore whether the OMI/non-OMI paradigm can improve outcomes by reducing treatment delays, the ongoing DIFOCCULT-3 trial (NCT06570759), a multicenter, randomized, open-label study enrolling 6,000 patients across 18 sites in Turkey, will evaluate *Queen of Hearts* AI-assisted ECG interpretation for the detection of high-risk patterns [20]. The primary composite endpoint is all-cause mortality and rehospitalization at one year; secondary endpoints include infarct size, left ventricular ejection fraction, wall-motion score index, and diagnostic accuracy for acute coronary occlusion. Recruitment began on October 1, 2024, with completion expected in October 2025 and one-year results anticipated in 2026.

## Conclusions

In a clinical context suggestive of myocardial ischemia, bifascicular block (BFB; RBBB + LAFB) should be regarded as a STEMI-equivalent pattern. It is typically associated with OMI due to proximal LAD or left main coronary artery occlusion and reflects extensive injury to both the myocardium and the conduction system. In RBBB, QRS-concordant STE in leads V1-V3 represents an unequivocal sign of STEMI. In our case, this pattern enabled early recognition of an LAD-culprit OMI and urgent reperfusion. The AI-based smartphone application *Queen of Hearts* (PMcardio) accurately recognized STEMI and the conduction abnormalities and suggested a reduced left ventricular ejection fraction, providing rapid confirmatory support at the point of care. BFB in the setting of ACS is an unfavorable prognostic marker, associated with ventricular fibrillation, cardiogenic shock, heart failure, and increased mortality. This high-risk ECG pattern should, therefore, be promptly identified and managed with immediate percutaneous reperfusion therapy. The modern era offers AI-powered diagnostic tools that can support clinicians in real-time ACS evaluation. While these technologies show promising accuracy and utility, their performance and generalizability cannot be inferred from a single case, and they remain vulnerable to misclassification, suboptimal ECG quality, and population-specific biases. These tools should be used judiciously as an adjunct and an aid, not a substitute, to expert ECG interpretation and clinical judgment.

## References

[1] Byrne RA, Rossello X, Coughlan JJ (2023). 2023 ESC Guidelines for the management of acute coronary syndromes. Eur Heart J.

[2] McLaren J, de Alencar JN, Aslanger EK, Meyers HP, Smith SW (2024). From ST-segment elevation MI to occlusion MI: the new paradigm shift in acute myocardial infarction. JACC Adv.

[3] Neumann JT, Sörensen NA, Rübsamen N (2019). Right bundle branch block in patients with suspected myocardial infarction. Eur Heart J Acute Cardiovasc Care.

[4] Lévy S (2018). Bundle branch blocks and/or hemiblocks complicating acute myocardial ischemia or infarction. J Interv Card Electrophysiol.

[5] Ricci F, Martini C, Scordo DM (2025). ECG patterns of occlusion myocardial infarction: a narrative review. Ann Emerg Med.

[6] (2025). Powerful Medical. Research. PMcardio: AI-powered ECG interpretation. http:////www.powerfulmedical.com/research/?utm_source=pmcardio_individuals&utm_medium=website&utm_campaign=impact_section.

[7] Bussink BE, Holst AG, Jespersen L, Deckers JW, Jensen GB, Prescott E (2013). Right bundle branch block: prevalence, risk factors, and outcome in the general population: results from the Copenhagen City Heart Study. Eur Heart J.

[8] Ibanez B, James S, Agewall S (2018). 2017 ESC Guidelines for the management of acute myocardial infarction in patients presenting with ST-segment elevation: the Task Force for the management of acute myocardial infarction in patients presenting with ST-segment elevation of the European Society of Cardiology (ESC). Eur Heart J.

[9] Widimsky P, Rohác F, Stásek J (2012). Primary angioplasty in acute myocardial infarction with right bundle branch block: should new onset right bundle branch block be added to future guidelines as an indication for reperfusion therapy?. Eur Heart J.

[10] Kurisu S, Inoue I, Kawagoe T (2007). Right bundle-branch block in anterior acute myocardial infarction in the coronary intervention era: acute angiographic findings and prognosis. Int J Cardiol.

[11] Strauss DG, Loring Z, Selvester RH (2013). Right, but not left, bundle branch block is associated with large anteroseptal scar. J Am Coll Cardiol.

[12] Rector G, Triska J, Ajene G (2023). Right bundle branch and bifascicular blocks: insensitive prognostic indicators for acute myocardial infarction. Curr Probl Cardiol.

[13] Horton CL, Brady WJ (2009). Right bundle-branch block in acute coronary syndrome: diagnostic and therapeutic implications for the emergency physician. Am J Emerg Med.

[14] Galcerá-Jornet E, Consuegra-Sánchez L, Galcerá-Tomás J (2021). Association between new-onset right bundle branch block and primary or secondary ventricular fibrillation in ST-segment elevation myocardial infarction. Eur Heart J Acute Cardiovasc Care.

[15] Myint Thu T, Hegazy A, Dancy L (2025). New right bundle branch block: a benign variant or an ominous sign?. Cureus.

[16] McLaren J. RBBB and occlusion MI (2025). Emergency Medicine Cases. McLaren J. ECG Cases 12: RBBB and occlusion MI. https://emergencymedicinecases.com/ecg-rbbb-occlusion-mi/.

[17] Shroyer S, Mehta S, Thukral N, Smiley K, Mercaldo N, Meyers HP, Smith SW (2025). Accuracy of cath lab activation decisions for STEMI-equivalent and mimic ECGs: physicians vs. AI (Queen of Hearts by PMcardio). Am J Emerg Med.

[18] Demolder A, Herman R, Vavrik B (2024). AI-powered smartphone application for detection of left ventricular systolic dysfunction using 12-lead ECG. Circulation.

[19] Herman R, Mumma BE, Hoyne JD (2025). AI-enabled ECG analysis improves diagnostic accuracy and reduces false STEMI activations: a multicenter U.S. registry [in press]. JACC Cardiovasc Interv.

[20] Aslanger EK, Aggül B, Yıldırımtürk Ö, Karabay CY, Meyers HP, Smith SW, Değertekin M (2025). A diagnostic paradigm shift in acute myocardial infarction: rationale and design of the DIFOCCULT-3 trial. JACC Adv.

